# How does English national end-of-life care policy impact on the experience of older people at the end of life? Findings from a realist evaluation

**DOI:** 10.1017/S1463423621000621

**Published:** 2021-10-27

**Authors:** Rhiannon Barker, Patricia Wilson, Claire Butler

**Affiliations:** 1 Research Fellow, Department of Health Services Research and Policy, Faculty of Public Health and Policy, London School of Hygiene and Tropical Medicine, London, UK; 2 Professor of Primary and Community Care, Centre for Health Services Studies, University of Kent, Canterbury, UK; 3 Clinical Professor of Palliative Medicine, Centre for Health Services Studies, University of Kent, Canterbury, UK

**Keywords:** end of life care, national policy, patient experience, realist evaluation, qualitative research

## Abstract

**Aim:**

To explore the extent to which national policy in end-of-life care (EOLC) in England influences and guides local practice, helping to ensure that care for older people at the EOL is of a consistently good quality.

**Background:**

Whilst policy is recognised as an important component in determining the effectiveness of EOLC, there is scant literature which attempts to interrogate how this happens or to hypothesise the mechanisms linking policy to better outcomes.

**Method:**

This article reports on the second phase of a realist evaluation comprising three case studies of clinical commissioning groups, including 98 in-depth interviews with stakeholders, meeting observation and documentary analysis.

**Findings:**

This study reveals the key contextual factors which need to be in place at micro, meso and macro levels if good quality EOLC for older people is to be achieved. The findings provide insight into rising local inequalities and reveal areas of dissonance between stakeholder priorities. Whilst patients privilege the importance of receiving care and compassion in familiar surroundings at EOL, there remains a clear tension between this and the medical drive to cure disease and extend life. The apparent devaluing of social care and subsequent lack of resource has impacted significantly on the way in which dying is experienced.

Patient experience at EOL, shaped by the care received both formally and informally, is driven by a fragmented health and social care system. Whilst the importance of system integration appears to have been recognised, significant challenges remain in terms of shaping policy to adequately reflect this. This study highlights the priority attached by patients and their families to the social and relational aspect of death and dying and shines a light on the stark disparities between the health and social care systems which became even more evident at the height of the Covid-19 pandemic.

## Introduction

Policies determining issues as complex as how patients should be treated at the end-of-life (EOL) are implemented within multifaceted and interacting social layers; influenced by a myriad of contextual factors from small scale interactions between patients and family or professionals to broader structural, societal and government processes. Whilst models and care pathways may appear theoretically sound, the way they play out when transposed onto a variety of different contexts may result in intended outcomes not being realised. The Liverpool Care Pathway (Neuberger *et al.*, 2013) was a case in point; a model for EOLC receiving broad-based professional support when it was introduced in the late 1990s and hailed as a means of bringing the best of hospice practice in palliative care into wider settings. Yet 15 or so years after it was first introduced, following adverse publicity, with patients’ families decrying the callous treatment they perceived their relatives to have received, the practice was withdrawn with a review attributing its failure in part to lack of resource provided to properly train staff (Neuberger *et al.*, 2013).

Globally people are living longer with more complex co-morbidities. In the UK, two-thirds of deaths now occur in individuals over the age of 75 years (Office for National Statistics, 2019). This changing demographic, together with the rise in numbers of the frail elderly, has signalled a call for the reorientation of palliative care, to incorporate not only specialists with a focus on specific diseases, but also greater requirements for those skilled in dealing with significant levels of need and complexity (Nicholson, 2017). As pressure on acute services grows, so does the imperative to find better ways of caring for the frail elderly in the community (NPELCP, 2015).

This article reports on findings from three London-based case studies undertaken as part of a broader piece of realist research, of which the scoping study has been previously reported (Barker *et al.*, 2020). The study set out to explore how effective English EOLC policy is ensuring services are in place that meets patients’ needs and requirements. Fieldwork was undertaken before the onset of the Covid-19 pandemic.

The study took a broad perspective, examining different system levels: micro, meso and macro. National EOLC policy is not well-defined, and commentators have pointed towards the myriad of guidance documents in circulation, some of unclear status and provenance (Centre for Health and Social Care Research, 2016). ‘Ambitions for Palliative Care’ (NPELCP, 2015) is accepted here as the most significant policy document to emerge in the last decade, presenting a framework of six ambitions for EOLC, namely to: individualise care; provide fair access; maximise comfort; coordinate care; ensure staff are prepared to care and facilitate community involvement. One of the central planks of the discourse espoused in national policy and interrogated in this article is that patient’s often want to die in familiar surroundings (their own home or nursing home) and that policy should support people’s choice to do so. Indeed, one of the current key performance indicators deemed indicative of the quality of EOLC focusses on the place of death. Yet, there remains a lack of clarity about where this should be. Whilst a number of surveys indicate that the majority of people express a preference for dying at home, this data has been called into question (Hoare *et al.*, 2015), particularly for older people as their condition deteriorates (Davidson and Gentry, 2013).

Realist evaluation (RE), suited to highly complex social situations (Greenhalgh and Papoutsi 2018), was chosen as an appropriate method to interrogate the questions posed in this study. A better understanding of how and why policies are implemented (political, social, economic influences) will strengthen the ability of actors to influence policy for the better (Gilson, 2012). Understanding how patients at the EOL and their carers perceive the care they receive, and developing a clear picture of their priorities, is key to arriving at effective, appropriate strategies to care for those who are dying. Policies are likely to be successful where there is alignment and cohesion between the aspirations of policy and the views of stakeholders (May *et al*., 2015).

The Economist Intelligence Unit (2015), which ranks the quality of death across forty countries internationally, includes the existence of national policy as a contributory factor in their appraisal system. The UK, in-part due to the demonstration of robust policy, is ranked at the top of the table. Yet, the process and mechanisms by which the existence of national policy leads to effective, equitable care remains opaque, and extreme variation in practice has been highlighted (CQC, 2016). This study sheds light on some of the mechanisms which led to policy being developed and implemented successfully.

## Aims and objectives

The study set out to explore how EOLC policy can help deliver equitable, good quality care to those at the EOL. Through a review of stakeholder priorities, it asked how these cohere with national policy and explored which contextual factors need to be in place to facilitate effective delivery.

## Method

**Figure 1.**
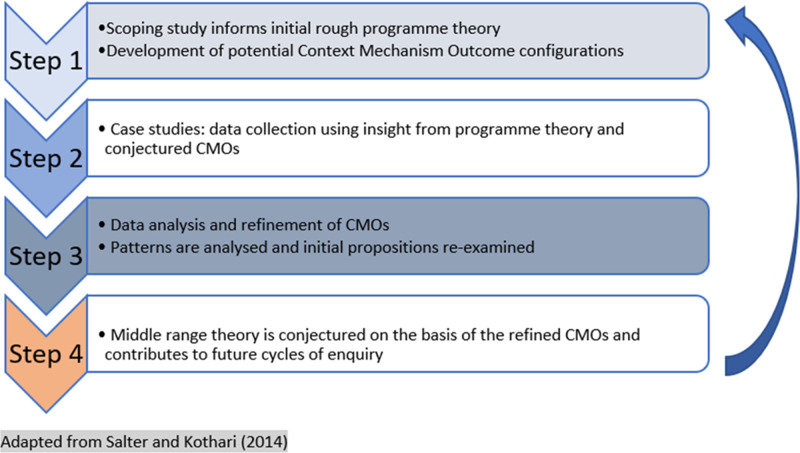
Stages of theory development

The study was theory-driven, following a RE methodology (Pawson and Tilley, 2004). Case studies were used to test and refine a set of conjectured mechanisms (developed during the scoping study) deemed responsible for achieving anticipated outcomes. Figure 1 illustrates the stages of theory development.

The aim was to shed light on what aspects of policy work – for whom – and in what circumstances (Pawson and Tilley, 2004). A realist approach assumes that outcomes and impacts are driven by a combination of context and mechanism – nothing works everywhere for everyone. Context mechanism outcome (CMO) configurations were developed within the course of research to describe how specific contextual factors work to trigger mechanisms which, in turn, produce outcomes (both intended and unintended).

The case studies provided an important tool to uncover the complexity of policy implementation, with findings being used to test and then revise the programme theory developed in the scoping study. Case studies do not set out to draw a representative sample from the population but rather to gather a detailed picture from those who have characteristics relevant to the phenomenon being investigated (Pope and Mays, 1995). Results from the case studies provide a fuller story illustrating how policy is interpreted by the various stakeholders, how this impacts on their ability to implement policy and in what circumstances it is most likely to be effective. A broader search for theories to help elucidate understanding and illuminate the significance of the findings for policy and practice offers further insight. The process advocated by realist evaluators, of drawing out ‘demi-regularities’ in research findings, which are represented in CMO configurations, leads to the conjecturing of ‘mid-range’ theory (MRT) and illuminates how findings may be used to inform practice. Theories are used to provide a new conceptual map that will enhance our understanding of how the mechanisms developed in the CMOs are triggered.

### Ethics

Ethics permission for the case studies was received from Stanmore Research Ethics Committee in October 2018 (IRAS project ID: 247 340), with additional permissions sought as necessary from relevant authorities (hospice, mental health and community trust, ambulance trust). Field work was completed between November 2018 and November 2019.

### Case study selection

At the time of planning fieldwork, during the Summer 2018, the NHS was undergoing significant transformation with the amalgamation of clinical commissioning groups (CCGs) into larger bodies referred to as sustainability and transformation plans (STPs). Within this shifting structural landscape, CCGs were primarily responsible for commissioning EOLC and therefore chosen to represent the most tangible governance structure, through which to organise case studies.

The selection of case study sites was guided by the proposed programme theory and informed by factors identified in the scoping study as being either indicative of, or likely to influence EOLC outcomes, presented in Table 1.

**Table 1.** Factors identified in scoping study (Barker *et al.*, 2020) as being indicative of, or likely to influence, EOLC outcomes

• Financial situation of local health economy
• Current performance of local services in relation to EOLC metrics (as determined by CQC and national data held by the National End of Life Care Intelligence Network)
• Demographic makeup of local population, including percentage of the population over the age of 75; ethnic breakdown; socio-economic profile
• Geographical location
• Urban or rural population
• Current governance arrangements for local health economy particularly in relation to the degree of service integration
• Presence of new models for EOLC/ways of working
• Level of development of sustainability and transformation plans (STP)/accountable care organisations/vanguards
• Strength and stability of local leaders
• Motivation, skills, expertise and support infrastructure of local specialised commissioners and clinicians

In line with RE methodology, the intention was to select three CCGs which would provide rich, detailed data to demonstrate some of the complexity around how EOLC policy impacts on outcomes, enabling us to use this data to test and develop our initial programme theory. Achieving a sample which was truly representative of EOLC practice in the 211 CCGs across England was untenable, yet basic stratification to reflect the contrasting characteristics likely to impact the experiences of EOLC was desirable. This, together with a nod to pragmatism and logistics, determined the final selection of three London based CCGs, chosen using the following criteria:

- Each case study was based within a different STP footprint to reflect the developing influence and policy of the broader geographical area;
- The sites represented a range of service models and EOL pathways;
- Sites reflected a range of performance outcomes and indicators for EOL services;
- Each CCG presented a unique set of demographics presenting contrasting local priorities;
- Key local staff was interested and motivated to participate in the research.

Whilst the three selected studies were London based and inevitably therefore reflect an urban and Southern bias, we contend that the themes identified provide valid insights which enable us to begin to interrogate and explicate the hypothesis developed in the scoping study and in so doing begins to piece together a complex and dynamic broader picture.

### Recruitment

Informants interviewed for the case studies were selected using non-probability, purposive sampling methods (Neuman, 2013). Professionals were stratified by job title, experience and area of interest, with efforts made to include clinical and non-clinical staff working across a range of settings with expertise in a variety of models of care. To this end, interviews were conducted across health, social care and voluntary organisations. Patients (over the age of 75 years) were stratified by setting and clinical prognosis and interviews were conducted in hospitals, care homes, hospices and patient homes. Particular efforts were made to access a sample of those living at home, who had been identified by their GP as likely to die in the next 12 months.

Across the three case study sites, a total of 98 in-depth interviews were conducted with the final sample shown in Table 2.

**Table 2.** Final sample of in-depth interviews completed across three case study sites

	Case study A	Case study B	Case study C
Non-clinical staff (roles included: CCG commissioners and accountants, care home and hospice managers, local authority leads, voluntary organisation managers, chaplains, adult therapy leads, volunteer coordinators, ambulance managers, out of hours service managers, CCG directors, academics)	6	12	10
Clinicians (roles included: GPs, health care assistants, palliative care nurses, community matrons, junior doctors, geriatricians, PC consultants, paramedics, locality clinical lead for EOL)	11	9	9
Patients (aged 75+, identified by clinicians as being likely to die in the next 12 months, cognitively able to enter into rational discussion, representing a mix of one or more different conditions)	5	8	9
Relatives (either linked to interviewed patients or who had relatives who were 75 years plus who they were currently caring for or who had died within the last year)	10	4	7

Notes from observations of meetings and secondary documentation such as Care Quality Commission (CQC) reports and minutes of relevant meetings were combined with interview data and uploaded into Nvivo – a software package that facilitates the managing of qualitative data. Mindful of improving data reliability, the study followed steps advocated by the RAMESES II study (Wong *et al.*, 2016) – providing an outline of suggested stages to be followed when using an RE method and Papoutsi *et al.* (Papoutsi *et al.,* 2021) for the building of programme theory.

Data were subjected to a number of coding iterations. Codes were categorised into higher level themes and categories and used to construct a series of CMO models which are used in RE to support the interrogation and development of theory.

## Results

Results from the case studies are presented in relation to the position in the broad social structure at which the identified causal mechanisms are seen to reside; micro, macro, meso.

**Figure 2.**
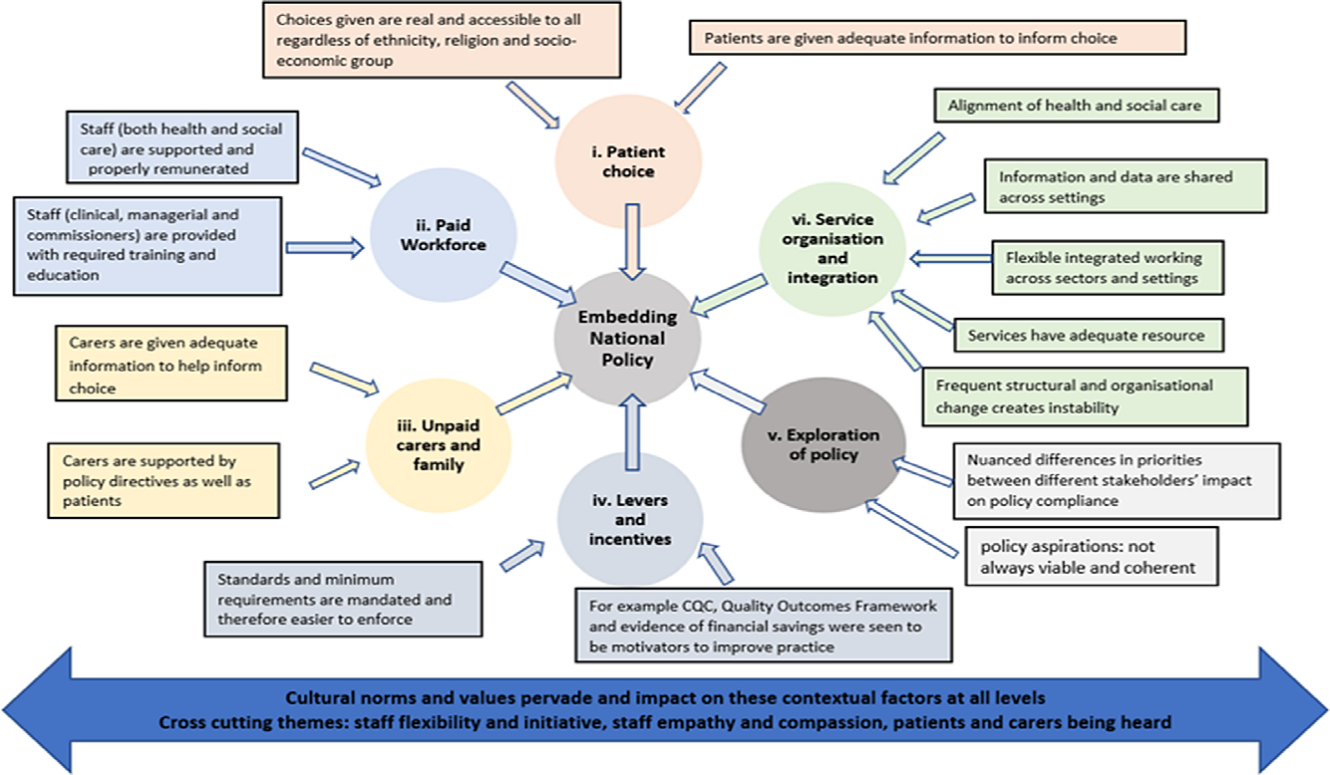
Contextual factors emerging from case studies that contribute to embedding EOLC national policy

Policy per se was valued by clinicians and managers in that it created a framework – laying out quality standards which could be set as aspirational markers. However, challenges to the implementation of policies were evident at all system levels. The context in which policy is implemented is highly complex with a number of causal processes at play at all levels. The wide range of contextual factors linked to EOL outcomes, emerging from the data, are outlined in Figure 2.

### Micro level factors

Patient narratives emphasised their desire to be treated with empathy and compassion and to die in a familiar environment with family or friends present. Patients prioritised the social and relational aspect of death and dying, with loneliness and isolation often being cited as key factors contributing to poor experience. Here, Elizabeth talks about her desire to remain in her care home:

*If my health gets worse I want to stay here and they know that…. I don’t want to go to hospital… If you go to hospital its unfamiliar – you’re surrounded by all sorts of new people. The care here is so good…in a hospital I think you would be told to get on with it. I wouldn’t get my Horlicks* (A.Patient.1).

Whilst many of those interviewed wanted to die at home, and policy privileges the enactment of this choice, the reality was that the services required to make this happen are often not available.

Links between patient experience and the support provided by front-line clinical staff working in the community, informal carers (family and friends) and paid ‘unskilled’ carers (usually employed through the local authority) was a strong emerging theme. The research indicated that the poor treatment and low morale of the social care workforce were reflected in poor patient experience. In this example, a patient’s son, Mark, voices the anger felt by many at the poor status and level of resource assigned to elderly social care.

*When my mum’s care package went out to tender from continuing health care (CHC) – they just go for the lowest quote. If you did that for a sick child, and they were sending carers who were falling asleep because they are so exhausted – there would be an outcry. But that’s fine for the elderly…. nobody really seems to care….* (A.Carer.3).

Despite the stress and anxiety of trying to find suitable support in what appeared to be a flawed system, many relatives expressed sympathy with the underlying problems:

*… the bottom line is that they’re paying them {social care staff} peanuts and not looking after them – what do they expect to get?* (A.Carer.7).

Although inevitably examples of good care were cited, the overriding impression was of a system where insufficient funding and support meant it was no longer fit for purpose. For relatives, being forced to witness the perceived disrespect with which their loved ones were treated, could be harrowing:

*There was one [carer] who didn’t even say hi to my mum – there was no engagement at all. I said to her one day it would be nice if you could at least speak to your client* (A.Carer.10).

The data indicated that for some patients, there was a tension between the medical (system level) drive to extend life and the desire to live the rest of their life, enjoying as much independence and social interaction with family and friends as their health would allow.

### Meso level

Gaps in 24/7 palliative care, shortage of home visiting from doctors and district nurses and lack of support to carers (paid and informal) were prevalent. There were also noticeable discrepancies in support, training and resource allocated to those commissioning EOL services. Here a hospital geriatrician describes what she saw as an unrealistic or exaggerated view of the palliative care (PC) services available in the community.

*I think there is an issue around being realistic about what faces a patient who wants to die at home and what we envisage happening from our positions inside the hospital … …. we sell a vision of a ‘great service’ – which is quite different from what people actually receive and experience… You begin to lose confidence in that aspiration to help to get people ‘out’ to die at home* (A.Clinician.11).

In addition, the findings show how the preferences and priorities of patients and relatives were influential in shaping the degree of motivation professionals showed towards enacting policy.

*It feels sometimes like if we ask people where they want to die – and they don’t give the system’s preferred choice, which is home, we just go back time and again until they give us the right answer* (A.Clinician.1).

Clinicians and service managers repeatedly spoke of the importance of being able to proactively raise questions with patients about preferred choice at EOL – yet many acknowledged that raising such a sensitive issue was challenging and required training. The majority of professionals in the research agreed that the existence of an advance care plan (ACP), created during EOL discussions, was important both to guarantee the improved patient experience and reduce inappropriate admissions to intensive care:

*Many patients, relatives and staff – only understand that someone is dying too late. They end up in ICU because no one had the time or the courage or the inclination to set a ‘ceiling of care’ with the family because it felt too difficult to do this* (B.Clinician.1).

Issues around choice were also complicated by evident differences in provision between local areas meaning that there was no one common set of ‘choices’ available. The ‘post-code lottery’ relating to care was widely acknowledged, with particular reference to marked differences in the way Fast Track Continuing Health Care was administered and also the availability of overnight palliative visiting services. Those service providers who worked across a number of different boroughs were particularly aware of the impact of inequitable services, as revealed in this comment from a manager of the ambulance service:

*There are such significant levels of variation between local areas that it can make it difficult to follow the wishes laid out in the Coordinate my Care (CMC) Plan*. (C.Non-Clinical.7).

Where staff were able to work flexibly, adapting their role to suit different situations, and where innovative integrated projects were being trialed that helped connect health and social care services, these were welcomed and the positive impact on patients was recognised. However, a number of significant challenges to joined-up working remain – particularly relating to the ability to share patient data across organisational and geographic boundaries. Here, a clinician voices the frustration about the numerous patient data systems:

*None of the systems talk to each other. The district nurse uses ‘System One’, the community nurse use CMC, someone else uses EMIS, we have everything on ‘charity log’ – nothing joins up … nothing*. (C.Clinical.2)

A number of other meso themes were prevalent including: organisational support; lack of routine training in communication techniques and identification of EOL; lack of support for commissioners to identify and implement evidence-based services; lack of flexibility in working across system boundaries.

### Macro level

At the macro level, the importance of integrated, joined-up systems where staff were enabled to work flexibly was emphasised, as was the urgent need to realign the health and social care sectors.

Numerous examples were cited to support the contention that clinical care was prioritised over relational care – reflected in the low pay and poor status of social care workers and by perceptions of staff in care homes and nursing homes, that they were seen as the ‘poor cousin of acute and primary care’. Although care home residents often have complex social and medical needs, they do not fall under NHS governance systems and for this reason have, over the years, fallen behind in terms of benefitting from routine support and training. One care home manager articulated the challenges faced in fighting to raise the quality and standards of what she saw as a pivotal, but largely undervalued, service:

*Too often people in social care are seen as second-class citizens …. In hospitals – when they are very stretched it’s understood that results in poor care – but people don’t seem to be so understanding of care homes…why are the staff in a care home different from the staff in hospital – or are they just given a lower value?* (A.Non-Clinical.2)

Whilst the professional rhetoric regarding integrated, joined-up working was strong, with a clearly stated vision to create seamless patient pathways, the reality rarely met the aspiration. For a number of clinicians, familiar with the local system, establishing better partnerships and joined-up working, was cited as their key priority:

*If my mother needed EOLC the one thing I would want is to ensure that the people looking after her were connected to and understand the different bits of the system* (B.Clinician.5).

**Figure 3.**
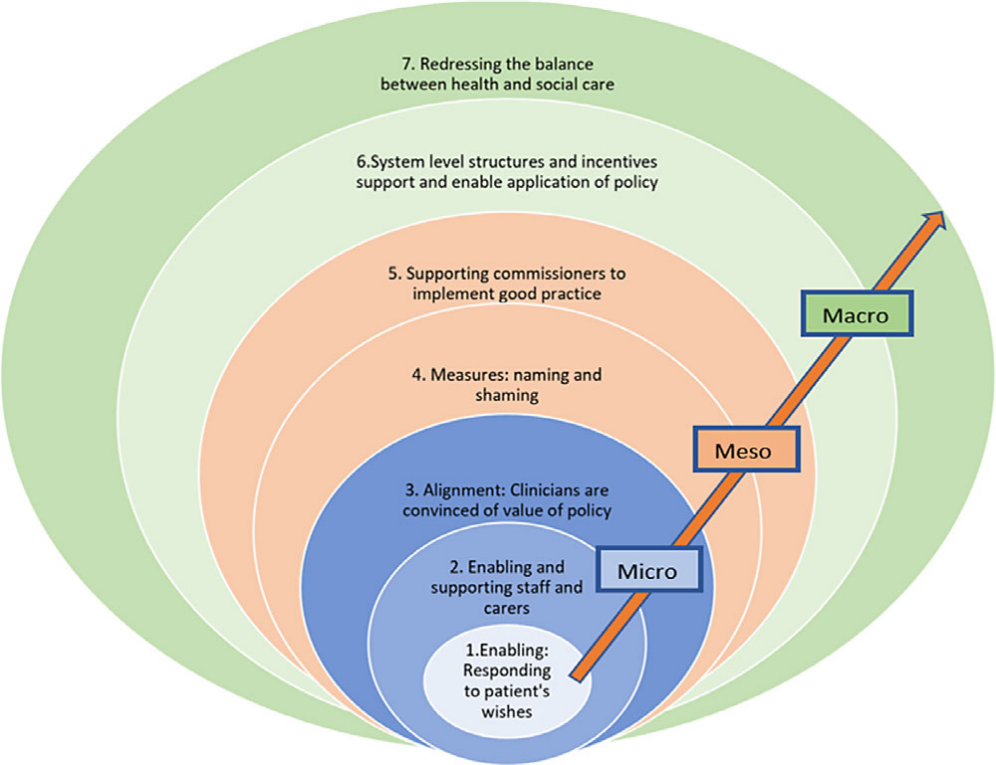
The seven proposed CMO configurations associated with enacting EOLC policy

The initial rough programme theory (RPT) emerging from the scoping study made the assumption that EOLC national policy helped to produce the intended outcomes (identified as consistent and good quality EOLC) without reference to the causal mechanisms behind the theory; the supposition being that the policy framework acts as a trigger to bring about a series of responses to put in place standardised services and monitor these against a set of aspirational statements. To help arrive at a revised programme theory, seven CMOs, represented in Figure 3, were identified to help explore and clarify the mechanisms at play in the implementation of EOLC at different system levels.

Based on the conjectured CMOs represented in Figure 3, a revised programme theory is proposed and presented in Table 3.

**Table 3.** Revised programme theory for the implementation of EOLC policy

**If the following contextual factors are in place:**
*At micro level*
• If the voices of patients are listened to and the system is able to provide choices which fit with patient preferences
• If carers and staff are supported so that they feel valued
• If EOLC policy is coherent and health professionals’ priorities and beliefs fit with the preferences of patients and their relatives
*At the meso level*
• If professionals (commissioners and clinicians) are provided with the necessary training and support and have access to the right forms of data on which to base decision making and if priorities held by the professionals align with those held by patients and carers
At the macro level
• If the system recognises that both clinical care and relational care are intrinsic determining factors in overall patient experience and provides joined up infrastructural support to enable effective; commissioning and provision of effective services
• If minimum standards can be set and levers identified to reduce current inequities in access to services
**Then:**
• Professionals can engage with policy and witness the value conveyed to patients, through following prescribed policy
• Stakeholders at different levels will act together, reflexively monitoring the impact of their work through observation and feedback from patients
Patients are more likely to have their wishes at the EOL recognised and fulfilled

## Discussion

This discussion focuses on findings emerging from the case studies, explores how these have informed the revised programme theory and asks what this adds to our understanding of how to improve the effectiveness of EOLC policy and practice. As in the findings section, implications at all system levels are considered, though the often overlooked voice of the patient is given particular attention.

Patient experience at the EOL, determined by the care received both formally and informally, is driven by a fragmented health and social care system characterised by significant discrepancies in local practice. The extreme variation evidenced across England suggests that local strategies and policy may be an influencing factor. One likely contributory factor in determining EOLC outcomes may relate to the different ways CCGs prioritise resources. The London Assembly Health Report (2016) noted sizeable variation in spending on EOLC across London with average spending varying across CCGs from £540 to £3,740 per death.

Whilst much of the challenge highlighted in this article resides at the broader macro level, there are nonetheless opportunities for commissioners and clinicians working at local levels to improve quality and reduce current inequities. Notably, it was evident that commissioners of EOLC took up new positions equipped with different levels of skill and knowledge. EOLC policy can provide the framework to help local commissioners identify the basic standards that need to be achieved and hence bring about more equitable service provision, but in order for this to happen, commissioners need to be equipped with skills, expertise and guidance. Routine provision of training for commissioners along with improved technical guidance and support to help identify both local needs and identify evidence-based services, may be an important step towards addressing the current discrepancies in local service provision. Achieving adequate and consistent health and social care support in the community, for those at the EOL, would provide clinicians with greater assurance and confidence that their patients, who wish to continue being cared for in the community, will have their needs met. This assurance is likely to improve clinician adherence to prescribed policy (for example, enabling patients to die at home) thus reducing the number of inappropriate emergency admissions into acute hospitals at the EOL.

The provision of training to both health and care professionals to help facilitate EOL conversations is another important part of enabling patient’s wishes to be met – and an integral part of encouraging this information to be collected is the need to improve the process for recording and sharing data between sectors and settings. Only once this is done will patients be held at the centre of their care, as policy espouses.

Data collected in this study suggested that the aspiration to achieve both greater integration and equity was yet to have a meaningful impact on day-to-day commissioning and operations. Access to pain relief, for example, was dealt with by the health system, whilst access to carers to provide emotional support, to feed and wash themselves or support to go to the toilet whilst living in the community, came from a different budget and was delivered from separate organisational settings. Decisions around budgets and priorities were made by separate commissioning arms, yet the way a patient experiences care is continuous and related to immediate and pressing needs. At a human level, the differentiation between health needs and social needs is artificial and leads to fragmented and inappropriate treatment.

A government policy paper (The Department for Business Energy and Industrial Strategy  2019) sets out a number of Grand Challenge Missions one of which relates to our ageing society and pledges that by harnessing the power of innovation people in England could:

*‘enjoy at least 5 extra healthy, independent years of life by 2035, while narrowing the gap between the experience of the richest and poorest.’*

Whilst the ambition to reduce inequality is to be applauded, the focus on length of life is worth noting. Should we continue to prioritise the extension of life whilst we continue to lack the resource to treat people who are dying with adequate levels of care and compassion? Budgets for health and social care are finite and spending in one sector has implications on what remains to be spent in others.

In the recent response to the Covid-19 pandemic, the government released a raft of policy directives targeted at a range of areas (Dunn *et al.*, 2020) covering both health and social care initiatives and including a frequent refrain from the Health Secretary relating to the ‘protective ring’ drawn up around care homes (Romei *et al.*, 2020). Yet Cowper (2020) writing in the HSJ considers how the gulf between the ‘two cultures’ of the NHS on the one hand and social care on the other has become increasingly polarised. As the pandemic spread in early March 2020, the government pledged to throw ‘whatever was needed’ at the NHS whilst social care remained largely ignored (Cowper, 2020) – indeed it was only as increasing numbers died in care homes that attention was turned to the sad plight of this forgotten sector. A Guardian editorial (Robert, 2020), proclaimed a verdict of ‘culpable neglect’ in regard to the government’s treatment of the care sector, citing: lack of routine testing; shortage of PPE; residents being isolated from families and routinely asked to sign ‘do not resuscitate orders’.

This research has highlighted the need to step back to reappraise how different parts of the health and social care system fit together and how we have arrived at our current set of priorities. Cuts in funding to social care over the last decade have exacerbated the inequities, with staff who care for the elderly at home being particularly poorly treated, underpaid and undervalued. Additionally, the potential to broaden responsibility for EOL, particularly promoting policies that enlist citizens to build local networks and mechanisms for supporting those at EOL, deserves closer investigation. Drawing on the notion of social capital (Putnam, 2000) a concept relating to the level of cohesiveness of the community and the strength of social ties, there has been a call (Kellehear, 2013; Sallnow *et al.*, 2016) to embrace a model of public health which places more emphasis on the social determinants of health and the influence of communities in the development of better health for all. A public health approach to palliative care seeks to build and operationalise social capital and encourage embedded civic action. Within this model, the focus is on empowering communities to support those dying, bringing to the fore expressions of a compassionate society and in so doing challenging the ‘professionalisation’ of EOLC (Kellehear, 2013).

The task confronted by politicians and commissioners, in dividing up the budget to support public services, is enormously difficult; with the need to consider a huge raft of moral, ethical, economic and social arguments. Yet unless the views of stakeholders at all positions in the social hierarchy, from those at the top level of government, commissioners, clinicians, families through to the frail elderly who are close to death, are fully considered – resulting policy, despite the rising sums of money invested, will fail to meet the needs and preferences of those it sets out to serve.

## References

[ref1] Barker R , Wilson P and Butler C (2020) Does national policy in England help deliver better and more consistent care for those at the end of life? *Journal of Health Services Research & Policy*. doi: 10.1177/1355819620914939 32228095

[ref27] Centre for Health and Social Care (2016) State of the Nations Report: Terminal Illness Care in England, Northern Island, Scotland and Wales. Shefflield. Sheffield Hallam University and Marie Curie.

[ref3] Cowper A (2020) Cowper’s cut: the two NHS cultures. Health Service Journal 5th April.

[ref4] CQC (2016) “A different ending”: addressing inequalities in end of life care. Overview report. CQC, Newcastle upon Tyne.

[ref5] Davidson S and Gentry T (2013) End of life evidence review. London, England, Age UK.

[ref6] Dunn P , Allen L , Cameron G and Alderwick H (2020) COVID-19 policy tracker: a timeline of national policy and health system responses to COVID-19 in England. London, England, The Health Foundation.

[ref7] Economist Intelligence Unit (2015) The 2015 quality of death index. Ranking palliative care across the world. London: The Economist Intelligence Unit.

[ref8] Gilson L (2012) Introduction to health policy and systems research. In Health policy and systems research: a methodology reader, 19. Geneva: Alliance for Health Policy and System Research, WHO, 39.

[ref9] Greenhalgh T and Papoutsi C (2018) Studying complexity in health services research: desperately seeking an overdue paradigm shift. BMC Med 16, 95. 29921272 10.1186/s12916-018-1089-4 PMC6009054

[ref10] Hoare S , Morris ZS , Kelly MP , Kuhn I and Barclay S (2015) Do patients want to die at home? A systematic review of the UK literature, focused on missing preferences for place of death. PLoS One 10, e0142723. 26555077 10.1371/journal.pone.0142723 PMC4640665

[ref11] Kellehear A (2013) Compassionate communities: end-of-life care as everyone’s responsibility. QJM: An International Journal of Medicine 106, 1071–1075. 24082152 10.1093/qjmed/hct200

[ref12] London Assembly Health Committee (2016) *End of life care in London*. https://www.london.gov.uk/sites/default/files/new_eolc_final.pdf. Accessed 11/10/2021.

[ref26] May C , Rapley T , Mair FS , Treweek S , Murray E , Ballini L , Macfarlane A , Girling M and Finch TL (2015) Normalization Process Theory On-Line Users’ Manual, Toolkit and NoMAD instrument. Available from http://www.normalizationprocess.org. Accessed 11/10/2021.

[ref14] Neuberger J , Guthrie C and Aaronovitch D (2013) More care, less pathway: a review of the Liverpool Care Pathway. London, England, Department of Health.

[ref15] Neuman WL (2013) Social research methods: Qualitative and quantitative approaches. London, England, Pearson Education.

[ref16] Nicholson C (2017) Frailty and End of life Care: Questions Creating Conversations. London, England, St Christopher’s Hospice.

[ref17] NPELCP (2015) Ambitions for palliative and end of life care: a national framework for local action 2015–2020. England, National Palliative and End of Live Care Partnership.

[ref18] Office for National Statistics (2019) National life tables, UK: 2016 to 2018. Available from: https://www.ons.gov.uk/releases/nationallifetablesuk2016to2018. Accessed 11/10/2021.

[ref19] Papoutsi C , Mattick K , Pearson M , Brennan N , Briscoe S and Wong G (2021) Interventions to improve antimicrobial prescribing of doctors in training (IMPACT): a realist review. Health Services and Delivery Research. 6(10). NIHR. Peninsual Medical School, University of Plymouth. 29489141

[ref20] Pawson R and Tilley N (2004) *Realist evaluation*. Paper funded by the British Cabinet Office. Available at: https://www.communitymatters.com.au/RE_chapter.pdf. Accessed 11/10/21.

[ref29] Pope C and Mays N (1995) Qualitative research: reaching the parts other methods cannot reach: an introduction to qualitative methods in health and health services research. BMJ, 311(6996), 42–45. 7613329 10.1136/bmj.311.6996.42 PMC2550091

[ref21] Putnam RD (2000) Bowling alone: the collapse and revival of American community. New York City. Simon and Schuster.

[ref28] Robert B (2020) UK healthcare regulator brands resuscitation strategy unacceptable. The Guardian, [online] Available at: https://www.theguardian.com/world/2020/apr/01/uk-healthcare-regulator-brands-resuscitation-strategy-unacceptable. Accessed 11 October 2021.

[ref22] Romei V , Plimmer G and Hughes L (2020) Hancock claim of ‘protective ring’ round care homes questioned. Financial Times. London, England. Financial Times. Available from: https://www.ft.com/content/6afb06d6-abd6-4281-ac16-74f500f096d0. Accessed online 11/10/2021.

[ref23] Sallnow L , Richardson H , Murray SA and Kellehear A (2016) The impact of a new public health approach to end-of-life care: a systematic review. Palliative Medicine 30, 200–211. 26269324 10.1177/0269216315599869

[ref24] The Department for Business E. and I.S. (2019) Policy paper: The Grand Challenge Missions. Available at: https://www.gov.uk/government/publications/industrial-strategy-the-grand-challenges/missions. Accessed 11/10/2021.

[ref25] Wong G , Westhorp G , Manzano A , Greenhalgh J , Jagosh J and Greenhalgh T (2016) RAMESES II reporting standards for realist evaluations. BMC Medicine 14, 96. 27342217 10.1186/s12916-016-0643-1 PMC4920991

